# The novel LESS (low-cost entrainment syringe system) O_2_ blender for use in modified bubble CPAP circuits: a clinical study of safety

**DOI:** 10.3389/fped.2024.1313781

**Published:** 2024-02-12

**Authors:** Andrew G. Wu, Sreyleak Luch, Tina M. Slusher, Gwenyth A. Fischer, Scott A. Lunos, Ashley R. Bjorklund

**Affiliations:** ^1^ Division of Critical Care Medicine, Boston Children’s Hospital, Boston, MA, United States; ^2^ Department of Pediatrics, Division of Pediatric Critical Care, Hennepin Healthcare, Minneapolis, MN, United States; ^3^ Department of Pediatrics, Chenla Children’s Healthcare, Kratie, Cambodia; ^4^ Department of Pediatrics Global Pediatrics, University of Minnesota, Minneapolis, MN, United States; ^5^ Department of Pediatrics, Division of Pediatric Critical Care, University of Minnesota, Minneapolis, MN, United States; ^6^ Biostatistical Design and Analysis Center, University of Minnesota, Minneapolis, MN, United States

**Keywords:** pneumonia, bubble CPAP, respiratory support, hyperoxia, global health, pediatrics, medical device

## Abstract

**Background:**

Bubble continuous positive airway pressure (bCPAP) is used in resource-limited settings for children with respiratory distress. Low-cost modifications of bCPAP use 100% oxygen and may cause morbidity from oxygen toxicity. We sought to test a novel constructible low-cost entrainment syringe system (LESS) oxygen blender with low-cost modified bCPAP in a relevant clinical setting.

**Methods:**

We conducted a clinical trial evaluating safety of the LESS O_2_ blender among hospitalized children under five years old in rural Cambodia evaluating the rate of clinical failure within one hour of initiation of the LESS O_2_ blender and monitoring for any other blender-related complications.

**Findings:**

Thirty-two patients were included. The primary outcome (clinical failure) occurred in one patient (3.1%, 95% CI = 0.1–16.2%). Clinical failure was defined as intubation, death, transfer to another hospital, or two of the following: oxygen saturation <85% after 30 min of treatment; new signs of respiratory distress; or partial pressure of carbon dioxide ≥60 mmHg and pH <7.2 on a capillary blood gas. Secondary outcomes included average generated FiO_2_'s with blender use, which were 59% and 52% when a 5 mm entrainment was used vs. a 10 mm entrainment port with 5–7 cm H_2_O of CPAP and 1–7 L/min (LPM) of flow; and adverse events including loss of CPAP bubbling (64% of all adverse events), frequency of repair or adjustment (44%), replacement (25%), and median time of respiratory support (44 h).

**Interpretation:**

Overall the LESS O_2_ blender was safe for clinical use. The design could be modified for improved performance including less repair needs and improved nasal interface, which requires modification for the blender to function more consistently.

## Introduction

Lower respiratory tract infections (LRTIs) continue to be the leading cause of death among children under five years old worldwide (1–4). This burden is particularly significant in low-middle income countries (LMICs) in Sub-Saharan Africa and Southeast Asia (3, 4). Respiratory support is key in decreasing mortality from LRTIs. Unfortunately, many respiratory support modalities, such as ventilators or non-invasive respiratory support devices, have limited availability in LMICs due to high cost, lack of trained staff for device maintenance and repair, reliance on electricity, and lack of specialized materials and parts (5).

**Figure 1:**
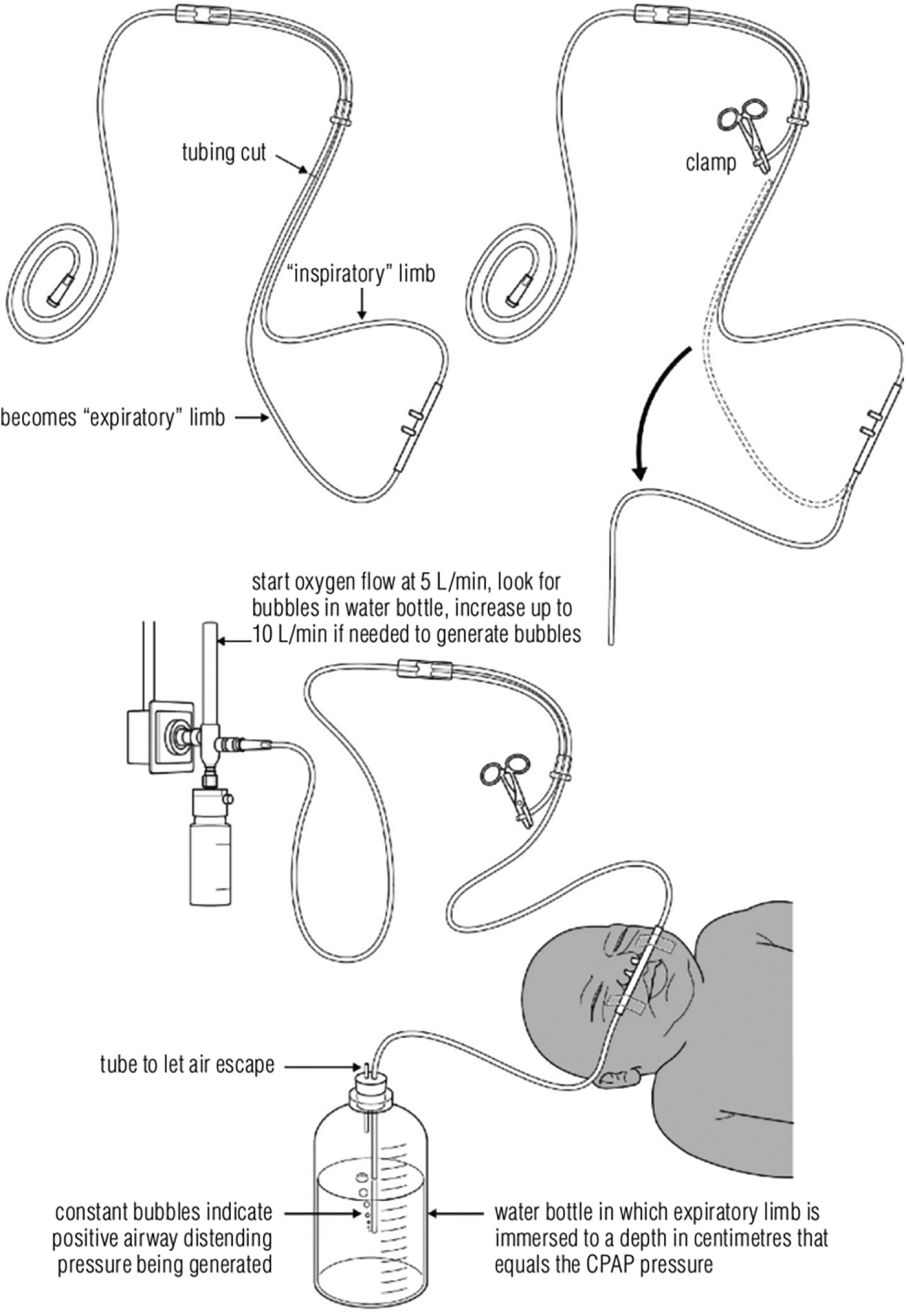
Bubble CPAP set-up. Reprinted with permission from the World Health Organization. A modified version of this circuit for older children was safety tested by Bjorklund, et al. that uses ear plugs to decrease nasal leak (19). CPAP, continuous positive airway pressure.

Continuous positive airway pressure (CPAP), a form of respiratory support, provides constant airway pressure stenting open alveoli at the end of exhalation, reducing work of breathing and improving oxygenation (6). Commercial CPAP devices cost thousands of dollars and require electricity. In the 1970's, an alternative form of CPAP, called bubble CPAP (bCPAP), was invented for neonatal use (7, 8). BCPAP uses an expiratory limb submerged in a water column which generates CPAP that approximates the pressure at the air water interface assuming minimal resistive pressure losses in the breathing circuit (9, 10). Unlike commercial CPAP, bCPAP circuits can be run without electricity using compressed air or oxygen. In multiple studies, bCPAP has been shown to successfully provide respiratory support and decrease neonatal mortality in both high-income countries (HIC) and LMICs (11–17). To address the burden of LRTI in resource-limited areas, a modified low-cost version of bCPAP has been developed using basic supplies readily found in hospitals in LMIC's (18). Promoted by the World Health Organization (WHO), this version has allowed hospitals to provide effective and safe respiratory support to young children at minimal cost [approximately 5 US dollars (USD) excluding oxygen costs] when the alternative is direct oxygen flow by nasal cannula without pressure support or spending thousands of dollars on an industry-level product (Figure 1).

One opportunity for advancement of this device is titration of the fraction of inspired oxygen (FiO_2_) delivered as this low-cost design usually uses 100% oxygen. High concentrations of oxygen can have harmful effects in the brain, lungs, heart, and eyes in neonates (20–22). Indeed, current guidelines for neonatal resuscitation recommend initially using 21% oxygen in term neonates, instead of 100% oxygen in order to avoid oxygen toxicity (23). Additionally, recent data have demonstrated significant associations between increased mortality and high oxygen levels among critically ill children outside of the neonatal period (24, 25). BCPAP has largely been tested in neonates although the burden of pneumonia remains highest in children less than five years old. There is a gap in low-cost, high quality respiratory support that minimizes oxygen toxicity for these children (26). Lastly, current methods of delivering variable concentrations of oxygen are generally expensive (approximately 1,000 USD) and not available to hospitals in LMICs (27).

**Figure 2:**
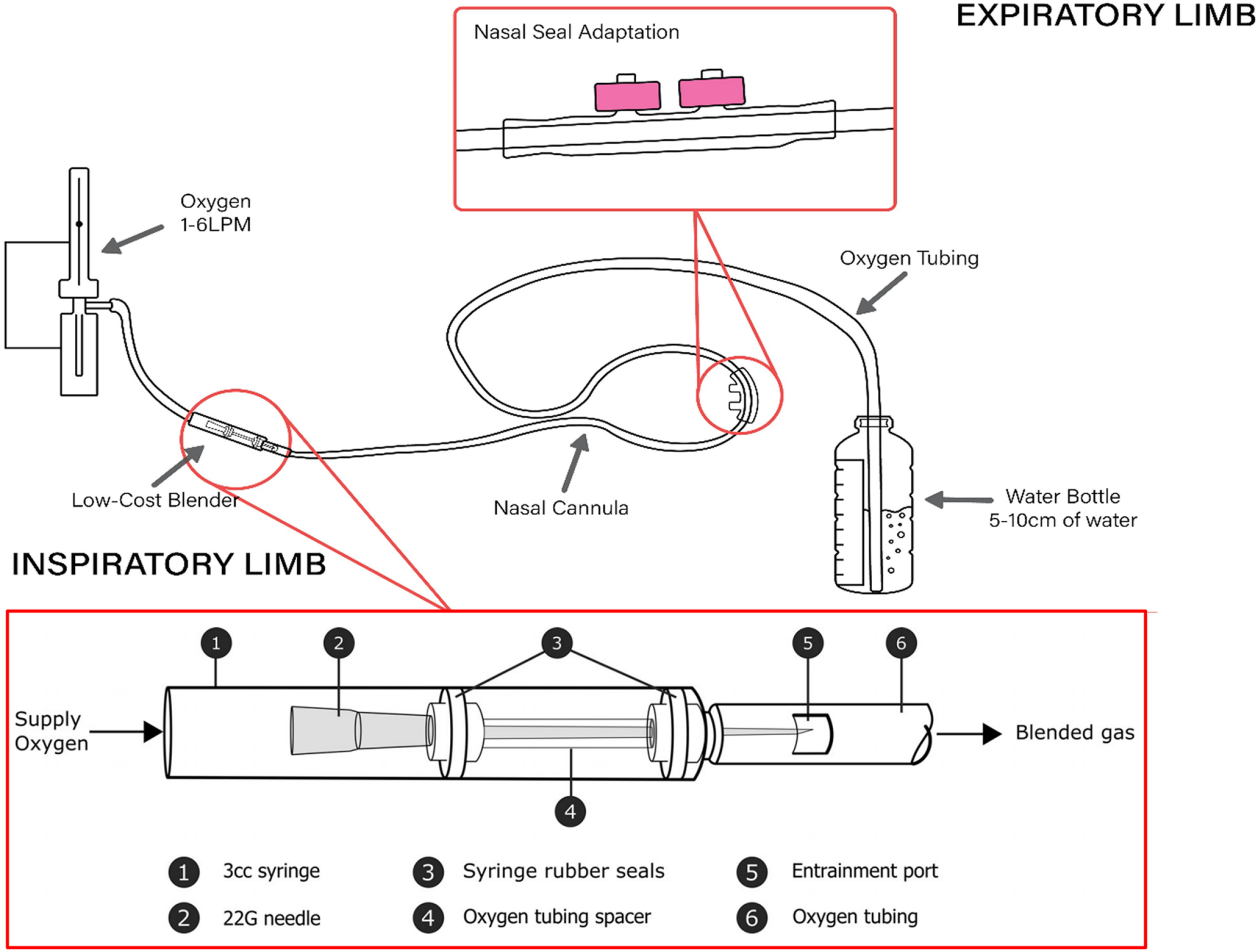
Bubble CPAP set-up with syringe oxygen blender in-line. Credit: Mara Halvorson.

In 2019, our team successfully developed an oxygen blender prototype utilizing jet mixing principles (Figure 2). By passing a high-velocity fluid jet (i.e., 100% oxygen stream) past quiescent fluid (i.e., 21% oxygen in room air), viscous shear forces promote mixing of the two fluids, allowing a titrated stream (i.e., <100% oxygen) of the two fluids to flow downstream to the patient. This blender, which we have called the LESS (Low-cost Entrainment Syringe System) O_2_ blender is designed to complement modified low-cost bCPAP circuit design and, like the bCPAP design, only requires common hospital supplies to build. The total cost of parts per circuit, which includes the blender and the modifications to the circuit, amounts to approximately 1.40 USD (excluding oxygen costs). Based on bench testing, the LESS O_2_ blender can decrease FiO_2_ to approximately 60%–70% and 40%–50% with 5 mm and 10 mm entrainment ports, respectively (28). We then tested construction among new users, which was performed in the USA and Cambodia, and found that it can be reliably constructed to generate mixed oxygen flows (29). The next step in development of this blender was to test its safety in a relevant clinical setting. We performed a clinical trial to evaluate the safety and feasibility of implementation of the LESS O_2_ blender with modified low-cost bCPAP in a children's hospital in rural Cambodia in 2022.

## Methods

### Device development

A multidisciplinary team (clinical and engineering) began working together in 2019 to design the blender. The LESS O_2_ blender utilizes the Venturi effect by funneling oxygen through a hypodermic needle that then exits near an entrainment port exposed to ambient air which facilitates blending of oxygen and room air in the delivered outflow oxygen tubing of the nasal cannula. It is designed to be constructed and assembled on site using two 3 ml syringes with rubber plunger stops, one 22-gauge hypodermic needle, oxygen tubing, super glue or tape, and a blade (i.e., scalpel/razor blade). During bench testing, delivered oxygen concentrations can be approximately 65% or 45% using the 5 mm entrainment port or 10 mm port respectively, and results were published in 2020 in the Journal of Medical Devices (28). For this study, we also used the SEAL-bCPAP modification, which minimizes leak at the nares (Figure 2) (19).

### Ethics and institutional approvals

The Institutional Review Boards (IRBs) at the University of Minnesota, Boston Children's Hospital, and Chenla Children's Hospital reviewed and approved this study.

The device was exempt from Food and Drug Administration review since the device is not intended for use in the USA. Additionally, the LESS O_2_ blender was given exemption from the determination protocols of the Department of Drugs and Food (DDF) in the Cambodian Ministry of Health given that the device has little to no sale potential, not intended to be sold in Cambodia; meets criteria for a low-risk device; and has no formal registration certificate from the country of export nor an associated company license given it is constructed on site.

### Role of funding source

The study sponsors did not have a role in study design; collection, analysis, or interpretation of data; writing of the report; nor the decision to submit the paper for publication.

### Study settings

The study took place at Chenla Children's Healthcare (CCH) located in Kratie, Cambodia, which is primarily rural and heavily affected by poverty. About one third of the residents live on <1 USD a day. Chenla Children's Healthcare runs a 30-bed pediatric ward, including six pediatric intensive care (PICU) beds and ten neonatal intensive care (NICU) beds. At the time of study initiation, there were five ventilators. There are bCPAP machines available but not enough to provide every child who is admitted for respiratory distress. Of note, in 2020 when the study was planned in pre-COVID pandemic, CCH had five bCPAP machines which increased to 18 in 2022 when the study was launched. Despite this increase, malfunctions in bCPAP machines are common and there are frequently more children requiring bCPAP than machines available.

### Device training and teaching

A team of Cambodian ICU nurses was trained on the study protocol and device construction via written instruction, in-person didactics, case discussions and simulated device building prior to study initiation. Constructed blenders that functioned as expected were kept for urgent use during study enrollment. The equipment and parts necessary to construct the low-cost bCPAP circuit and the syringe blender were provided.

### Study design

This study was designed as a prospective cohort feasibility and safety pilot study. Patients were enrolled from March 2022 to March 2023. Participants were eligible if they met all criteria in Table 1.

**Table 1:** Inclusion and exclusion criteria.

Inclusion criteria	Exclusion criteria
• 5 years of age or younger AND • Admission diagnosis of a lower respiratory tract infection (LRTI) such as pneumonia or bronchiolitis ◦ For neonates (under 1 month of age), respiratory distress syndrome, transient tachypnea of the newborn, and meconium aspiration qualified for inclusion AND • Respiratory distress upon presentation to the hospital, defined as cough or trouble breathing plus at least one of the following ◦ <92% despite low flow oxygen ◦ Central cyanosis ◦ Tachypnea ◦ Chest indrawing ◦ Nasal flaring ◦ Grunting ◦ Head nodding ◦ Convulsions ◦ Lethargy ◦ Inability to breastfeed or drink AND • No bubble continuous positive airway pressure (bCPAP) machine is available for immediate use	• History of asthma • Upper airway obstruction • Diaphragmatic hernia • Pneumothorax • Acute Glasgow Coma score <4 • Cleft Palate • Cyanotic heart disease • Congenital lung disease • Bleeding disorders • Imminent death within 2 h • Have had abdominal or thoracic surgery

Eligible patients must have been five years old or younger, have an admission diagnosis of LRTI, and have respiratory distress. If a bCPAP machine was immediately available at the time of enrollment, the patient was not enrolled. If the patient presented with any of the exclusion criterion, the patient was not enrolled.

Our target sample size was 50 to obtain preliminary feasibility and safety data. Written consent from parents/guardians was obtained prior to enrollment.

Enrolled patients were initiated on modified low-cost bCPAP without the LESS O_2_ blender (Figure 1). Once the patient achieved respiratory stability, which was defined as having an oxygen saturation >90% and capillary refill <2 s, the low-cost bCPAP circuit was replaced with the LESS O_2_ blender circuit. Though LESS O_2_ blender circuits from the training were available to use, nurses were encouraged to create new syringe blender bCPAP circuits to maximize cleanliness. Circuits were single-patient use. The TAL respiratory score was obtained on enrollment to assess the severity of respiratory distress before initiation of respiratory support (30).

After the LESS O_2_ blender circuit was initiated, study nurses assessed the device 30 min later to confirm device function and again at one hour to assess for clinical failure. Nurses evaluated device function every two hours, including measurement of the FiO_2_ level at the nasal cannula via an oxygen analyzer placed over one nasal prong (Analytical Industries Palm D Oxygen Analyzer). Vitals and exam findings were documented every four hours. Due to limited lab availability, the only labs obtained for study purposes were a complete blood count, malaria rapid diagnostic testing (RDT), and a bedside blood gas and electrolyte analyzer, all of which are standard of care at the site. No malaria RDT was required for neonates. Titration of the FiO_2_ and CPAP levels were left to the discretion of the ordering physician with use of continuous pulse oximetry, though training was provided for which entrainment port size could be utilized.

If the blender circuit was found to be malfunctioning (e.g., lack of bubbling, twisted tubing, oxygen leak) the patients were assessed for stability. Then the study nurse would troubleshoot the device. If troubleshooting required removal of the circuit from the patient, the patient was placed on low-cost bCPAP without the blender until the blender circuit was repaired or replaced.

### Outcomes

The primary study outcome was clinical failure within one hour of changing to the LESS O_2_ blender circuit. Clinical failure was defined as intubation, death, transfer to a higher level of care (i.e., another hospital), or two of the following:

• Oxygen saturation <85% after 30 min of treatment
• Signs of respiratory distress, including indrawing, tracheal tugging, nasal flaring, or grunting
• Partial pressure of carbon dioxide ≥60 mmHg and pH <7.2 on a capillary blood gas

The secondary outcomes of the study included the following:

• Number of times no bubbling was noted and/or blender/bCPAP circuit required repair or replacement
• Duration of respiratory support in hours
• Adverse events related to the blender circuit (described below)
• Outcome of hospitalization

Outcomes related to functioning of the LESS O_2_ blender included the following:

• Size of entrainment port
• CPAP level
• Oxygen concentration (FiO_2_) of nasal cannula outflow
• Flow of oxygen from tank

Adverse events were documented in detail and were defined as either grade I or grade II, with the latter considered serious adverse events (SAE) and characterized by associated clinical decline. Adverse events could be related to the modified circuit (nasal injury, nose bleeding, aerophagia, pneumothorax, device fragmentation at circuit connections) or the blender itself (loss of CPAP, oxygen leak, device fragmentation).

### Statistical analysis

Descriptive statistics (means and standard deviations or medians and IQR for continuous variables; counts and percent for categorical variables) were used to summarize patient demographics and outcomes. The clinical failure rate was estimated along with an exact binomial 95% confidence interval. Spearman correlation coefficients, Kruskal–Wallis tests and Wilcoxon rank sum tests were used to compare variables of interest with duration of respiratory support, number of repairs, and number of replacements. A linear mixed effect model was used to compare mean FiO_2_ between port sizes while controlling for CPAP level and flow. A random patient effect was included to account for multiple tracking measures per patient. SAS V9.4 (SAS Institute Inc., Cary, NC) was used for the analysis.

## Results

**Figure 3:**
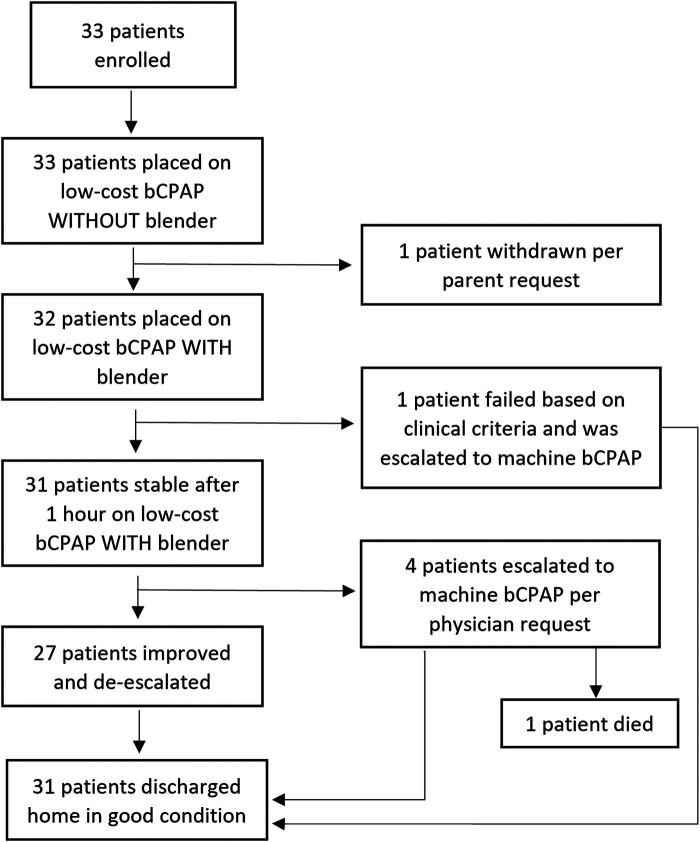
Flowchart of enrollment. bCPAP, bubble continuous positive airway pressure.

Thirty-three patients were enrolled (Figure 3). The majority were male (66.7%), less than three months old (76%), and term gestation (88%). At enrollment, 91% were deemed stable, and 65.2% had moderate respiratory distress (Table 2). The mean respiratory rate on admission was 52 breaths per minute (SD 8) and the mean oxygen saturation on admission was 93.5% (SD 6.6%).

**Table 2:** Baseline demographics and characteristics.

	*N* = 33
Gender, *n* (%)	
Female	11 (34·4)
Male	22 (68·8)
Age, *n* (%)	
<1 months	15 (45·5)
1–3 months	10 (30·3)
4+ months	8 (24·2)
Stable on enrollment, *n* (%)	30 (90·9)
Gestational age at birth, *n* (%)	
30–36 weeks	4 (12·1)
37–38 weeks	4 (12·1)
39 weeks-Full term	25 (75·8)
Weight (kg), mean (SD)	4·6 (2·0)
Oxygen saturation, mean (SD)	93·5 (6·6)
Respiratory distress based on Tal Score, *n* (%)	
Mild respiratory distress	
Moderate respiratory distress	11 (33·4)
Severe respiratory distress	22 (66·6)
	0 (0·0)
Pediatric Early Warning Sign (PEWS) Score, *n* (%)	
Mild	28 (84·8)
Moderate	5 (15·1)
Severe	0 (0·0)

Data are demonstrated as count and proportions unless otherwise noted.

One patient was withdrawn due to the parental request to leave against medical advice and was never placed on the LESS O_2_ blender, leaving 32 participants who used the LESS O_2_ blender circuit. One patient met criteria for the primary outcome of clinical failure 40 min post initiation of the LESS O_2_ blender (3.1%, 95% CI = 0.1%–16.2%). Of note, this patient who failed did not receive 30 min of treatment prior to escalation due to acuity of the clinical situation and physician clinical judgement (i.e., was immediately escalated and eventually intubated). One patient who died during the hospitalization from septic shock (not study related) was escalated to machine bCPAP after eight hours on the LESS O_2_ blender circuit due to hypoxemia and central cyanosis felt to be due to disease progression. The remaining 31 patients, including the one who failed the blender, were eventually discharged home. Four patients were escalated to machine bCPAP on request of the physician and these transitions occurred after completing the first hour on the LESS O_2_ blender. Capillary gases were obtained on all participants as per hospital protocol, but no patients met clinical failure criteria based on the results.

Most adverse events recorded were due to lack of bubbling, accounting for 64% (18/28 events in 19 patients) of the events (Table 3). Lack of bubbling was caused by leak in the circuit requiring tape or straightening of the entrainment port; incorrect entrainment port size; the expiratory limb accidentally retracting from the water; using nasal prongs too small for the child; and child crying (i.e., leak from mouth). No adverse events were documented as severe. Fourteen patients had their blender circuit repaired at least once with 10/14 requiring only one repair. Examples of repairs included reinforcing circuit leaks with tape or glue, realigning needle, untwisting entrainment port, and re-inserting expiratory limb in the water or adding more water. Eight patients required circuit replacement, with 6/8 requiring only one replacement. Most replacements were done for lack of bubbling, followed by troubleshooting without success and then replacement. Experiencing more repairs or replacements were not significantly correlated with weight, age, gender, gestational age, or TAL respiratory score. There were two nasal septal injuries which were grade I and described as “redness of the nose”.

**Table 3:** Summary of outcomes and adverse events.

	*N* = 32
Clinical failure, *n* (%)	1 (3·1)
Number of times no bubbling/Continuous positive airway pressure (CPAP) failure noted, *n* (%)	
0	15 (46·9)
1	9 (28·1)
2	4 (12·5)
3	4 (12·5)
Number of times blender required repair, *n* (%)	
0	18 (56·3)
1	10 (31·3)
2	3 (9·4)
3	1 (3·1)
Number of times blender required replacement, *n* (%)	
0	24 (75·0)
1	6 (18·8)
2	1 (3·1)
3	1 (3·1)
Number of patients who experienced adverse event(s), *n* (%)	19 (59·4)
Loss of CPAP, *n* (%)	15 (46·9)
Device fragmentation of blender, *n* (%)	5 (15·6)
Nasal septal injury, *n* (%)	2 (6·3)
Other, *n* (%)	6 (18·8)
Duration of respiratory support (hours)	
Median (IQR)	44·0 (13·6–78·0)
Hospitalization outcome, *n* (%)	
Die	1 (3·1)
Discharge	31 (96·9)

Thirty-two patients are represented here instead of 33 as one patient withdrew from the study before initiation of the LESS O_2_ blender. Data are demonstrated as count and proportions unless otherwise noted.

The median duration of respiratory support was 44 h (IQR 13.6–78.0 h). The median (IQR) time to CPAP loss was 10.5 h (4.0–28.8). The median duration of support among children who experienced loss of CPAP was the same as the median duration of support among those who did not experience loss of CPAP (44 vs. 44 h, *p* = 0.72). Similarly, duration of support did not differ statistically across weight (*p* = 0.27), gestational age (*p* = 0.83), age at admission (*p* = 0.89), gender (*p* = 0.37), presence of a danger sign (e.g., inability to feed, convulsions, or decreased level of consciousness) (*p* = 0.98), or meeting sepsis criteria (*p* = 0.11).

**Figure 4:**
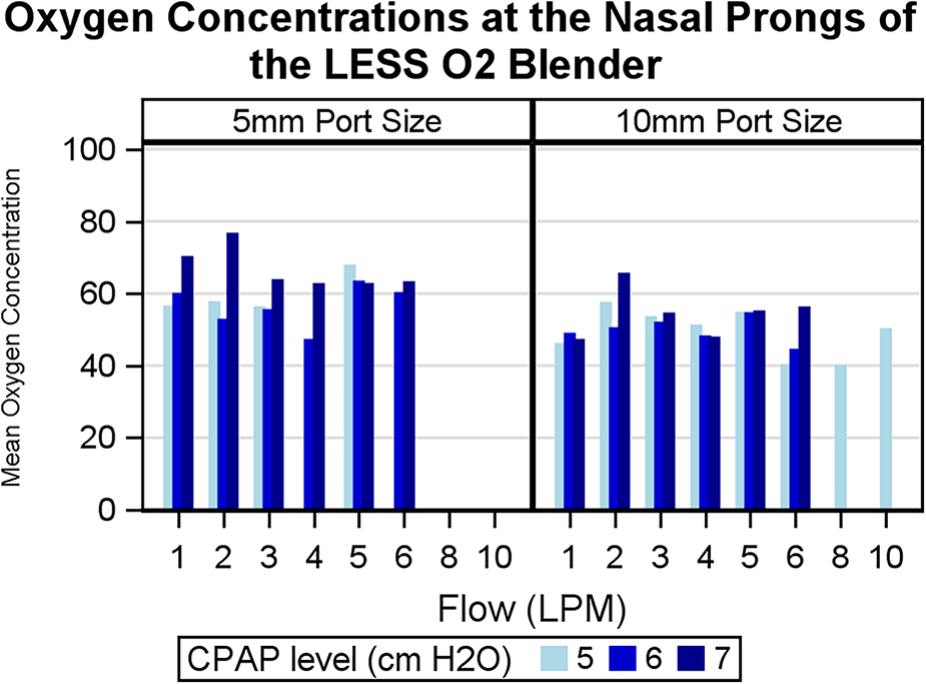
Histogram displaying measured oxygen concentrations of the gas mixture at the nasal prongs of the LESS O_2_ blender circuit. The left half demonstrates measurements using a 5 mm entrainment port and the right demonstrates measurements using a 10 mm entrainment port. Oxygen concentrations are graphed across flows in liters per minute (LPM) and by CPAP level in cm H_2_O. CPAP, continuous positive airway pressure.

The average FiO_2_ delivered by the syringe blender circuit with any entrainment port size was 54.1% (SD 8.7%). The average minimum FiO_2_ was 47.5% (SD 8.6%) and the average maximum was 61.9% (SD 12.1%). The 10 mm entrainment port was used more often than the 5 mm (70% vs. 30% of recorded port lengths, respectively). Figure 4 displays the FiO_2_ generated as it varied by flow and CPAP level used. The average FiO_2_ of the 5 mm port was 59.0% and the average FiO_2_ of the 10 mm port was 52.1% (*p* < 0.0001).

## Discussion

Our study sought to test the safety and feasibility of a novel, constructible oxygen blender designed to be used with low-cost, modified bCPAP with the SEAL-bCPAP modification. The burden of pediatric mortality from pneumonia disproportionately occurs in LMICs where respiratory support technology can be very limited. Our device is an additional tool for practitioners caring for sick children in the most resource-limited settings as it can provide both respiratory support with less oxygen toxicity and without electricity. Overall, we found our device to be safe based on low clinical failure among the population tested, and it provided mixed oxygen concentrations to minimize hyperoxia, though circuit leaks required attention. Nurses were available to troubleshoot the device and continuous pulse oximetry was always used. On average, patients were found to have moderate respiratory distress on enrollment based on increased work of breathing and to a lesser degree, hypoxemia.

A similar study was conducted in a 2016 study in India using an aquarium air pump with bCPAP to deliver mixed oxygen concentrations of 42%–51% (31). No patients decompensated with blender use. Of note, this study has a couple of key differences from our proposed study. First, our study enrolled patients up to 5 years versus only neonates in the Indian study. Second, our device does not require electricity or purchase of a pre-made device, whereas this study in India required a reliable source of electricity and a specific type of aquarium air pump. In recent years, there have also been other groups, such as PATH and Vayu Global Health Innovations, developing low-cost oxygen blenders designed for bCPAP, which is excellent as these open up options for practitioners in LMICs (32, 33). Of note, these devices are usually either 3D-printed or injection molded in the HIC and then transported. In contrast, our blender can be constructed on site with common medical supplies, which provides an avenue for the most resource-restricted areas to have access to methods of reducing oxygen concentrations without additional funding or dependency on high-resource countries for supplies.

We originally hypothesized that the most anticipated concern with the circuit would be leaks of oxygen, leading to loss of bubbling and therefore CPAP, due to the multiple connections required in the blender's construction. Based on our findings, these leaks were largely due to the modified nasal interface. Our design requires the “implantation” of nasal prongs onto oxygen tubing, which allows for CPAP to occur since the tubing is larger bore. However, this design introduces potential leak at the site of implantation. An additional issue was that oxygen tubing is relatively inflexible (compared to smaller, more compliant nasal cannula tubing), which led to prongs repeatedly coming out of the nares when used with a physically active child. Many bCPAP circuits use large bore, high resistance tubing with short binasal prongs or a mask (9). Taken together, the LESS O_2_ blender provides the advantage of being constructed on site and therefore an element of self-sufficiency. However, the constructible aspect also introduces a level of imprecision that demands close monitoring of the presence of bubbling. This also translated to increased vigilance and time spent by the nursing and physician teams to monitor the patients very closely. Therefore, use of this device requires enough medical staff to be present and attentive. Of note, six nurses identified by the on-site Cambodia-based investigator (SL) as having exceptional skill in constructing the blenders were deemed “superusers” and tasked with building the circuits if one was needed urgently (i.e., if replacement was required) or if any troubleshooting problems arose. This was a strategy we implemented in order to mitigate mechanical device problems after DSMB review at the mid-point of the study. This device is not intended to supplant a precision manufactured device. The LESS O_2_ blender circuit is designed for use where the only other options are either low flow nasal cannula or low-cost, constructible bCPAP using 100% oxygen.

In a previous study, the relationships among FiO_2_, flow, and CPAP level in our device were explored. Among circuits with a 10 mm entrainment port, the FiO_2_ remained constant across flows 3–10 LPM and varied slightly (±3 points) across CPAP levels (1–8 cm H_2_O). Conversely, circuits with a 5 mm entrainment port delivered FiO_2_'s that were constant unless flows were <4 LPM, at which point the FiO_2_ would typically increase by 5–10 points (28). This occurred due to a lower pressure gradient with lower flows, leading to reduced entrainment of room air in the gas mixture. We noted that higher CPAP levels correlated with FiO_2_, which was likely from increased back pressure resulting in less entrainment of room air as well. In this clinical study, we found similar patterns—specifically FiO_2_ largely remained constant across flow rates and higher CPAP levels were associated with higher delivered FiO_2_ in the circuits with only the 5 mm port. For these reasons, we advise practitioners using the LESS O_2_ blender be aware that as the CPAP is increased, the FiO_2_ will likely increase as well. We limited our CPAP to ≤8 cm H_2_O. Flow should be increased just enough to generate gentle bubbling throughout the respiratory cycle (34).

Limitations include the inability to measure either a reduction in oxygen toxicity (i.e., free radicals) or decrease in mortality, both impractical for our purposes. Additionally, our study population was largely term infants with mild-to-moderate respiratory distress. Patients with severe respiratory distress or those >8 months old may not be adequately supported with this device. The target sample size of 50 was not achieved due to lack of enrollment by the pre-determined study end time of March 2023. Despite estimated calculations of an appropriate timeline to reach the target sample size, the COVID19 pandemic caused significant delays in both the USA and Cambodia leading to a delayed study start in 2022, at which time the hospital had acquired many more bCPAP machines which hindered higher enrollment rates. The end time of March 2023 (i.e., one year study duration) was limited by funding. Despite these barriers, the enrolled sample size was adequate and appropriate to provide valuable data about feasibility and safety.

## Conclusion

The ability of the LESS O_2_ blender to reliably deliver blended oxygen and reduce the burden of hyperoxia is a major benefit of its use. Leaks noted in the circuit were the most frequent issue. If the bubbling is not present constantly (34), practitioners should inspect for leaks at the nasal cannula, the blender housing connections, and/or the entrainment port. The instructions for device use and construction are included with this publication (See Supplementary Materials). The authors intend for the LESS O_2_ blender to be open source and available to learn, use, and modify as needed by providers around the world.

As next steps, our team plans on improving the nasal interface and identifying ways to modify the oxygen tubing such that leaks will be minimized but that also less handling by the medical staff during use may be required.

The LESS O_2_ blender is ready to use in the field for practitioners caring for children in very resource-limited settings, such as areas without reliable electricity, compressed air, limited international bandwidth, or restricted funding. We emphasize that medical staff should remain attentive to the presence of bubbling and the integrity of the device if it is to be used and that continuous oximetry should be placed on the patient. Additionally, the instructions and guidelines included in this publication should be carefully read before deciding to use (See Supplementary Materials). Currently, there is no low-cost oxygen blender for bubble CPAP that can be assembled on site to our knowledge, allowing the LESS O_2_ blender to address this current critical gap.

## Data Availability

The original contributions presented in the study are included in the article/**Supplementary Material**, further inquiries can be directed to the corresponding author.
