# Supplementary material for: The novel LESS (low-cost entrainment syringe system) O2 blender for use in modified bubble CPAP circuits: a clinical study of safety
Source: Front Pediatr. 2024 Feb 12;12:1313781. doi: 10.3389/fped.2024.1313781 (PMC10894966; doi:10.3389/fped.2024.1313781)
Supplement: Supplementary file 1 [file Datasheet1.docx]

**Low-cost Entrainment Syringe System (LESS) O_2_ Blender**

**for Low-Cost Bubble CPAP Use**

**in Resource-Limited Settings**

**Instruction Guide for Construction and Use**

**Written by**

**Jared Floersch, Adam Keester, Andrew Wu, Ashley Bjorklund**

**Illustrations by**

**Jared Floersch, Adam Keester, Mara Halvorson**

**Last Updated 8/24/2023**

**Table of Contents:**

Overall Circuit…………………………………………………………………………………..…………………………..3

Supplies Needed………………………………………………………………………………..………………………….3

Instructions for Assembly………………………………………………………………………..………………………..4

Instructions for Use………………………………………………………………..……………………………………..11

Preventing Complications………………………………………………………………..……………….………..……12

Troubleshooting………………………………………………………………..…………………………………………13

References………………………………………………………………………………………………………………...14

Contact and Support……………………………………………………………………………………………………..14

Overall Circuit

Below is a diagram detailing how the final device looks when assembled. Keep this in mind when building the device.


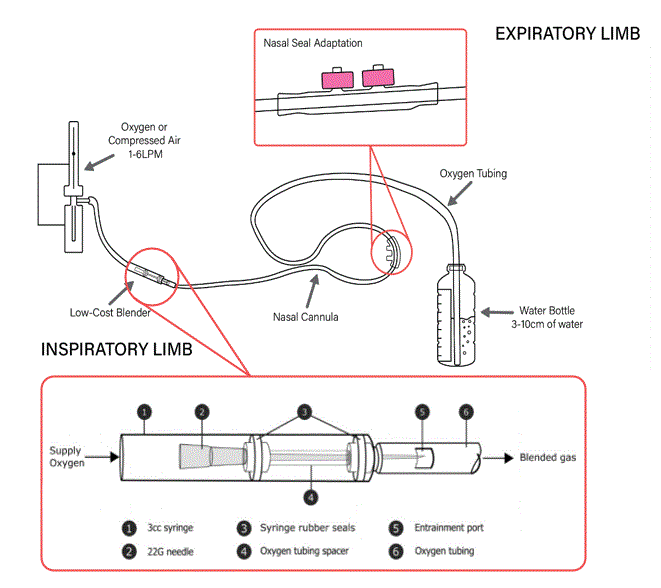

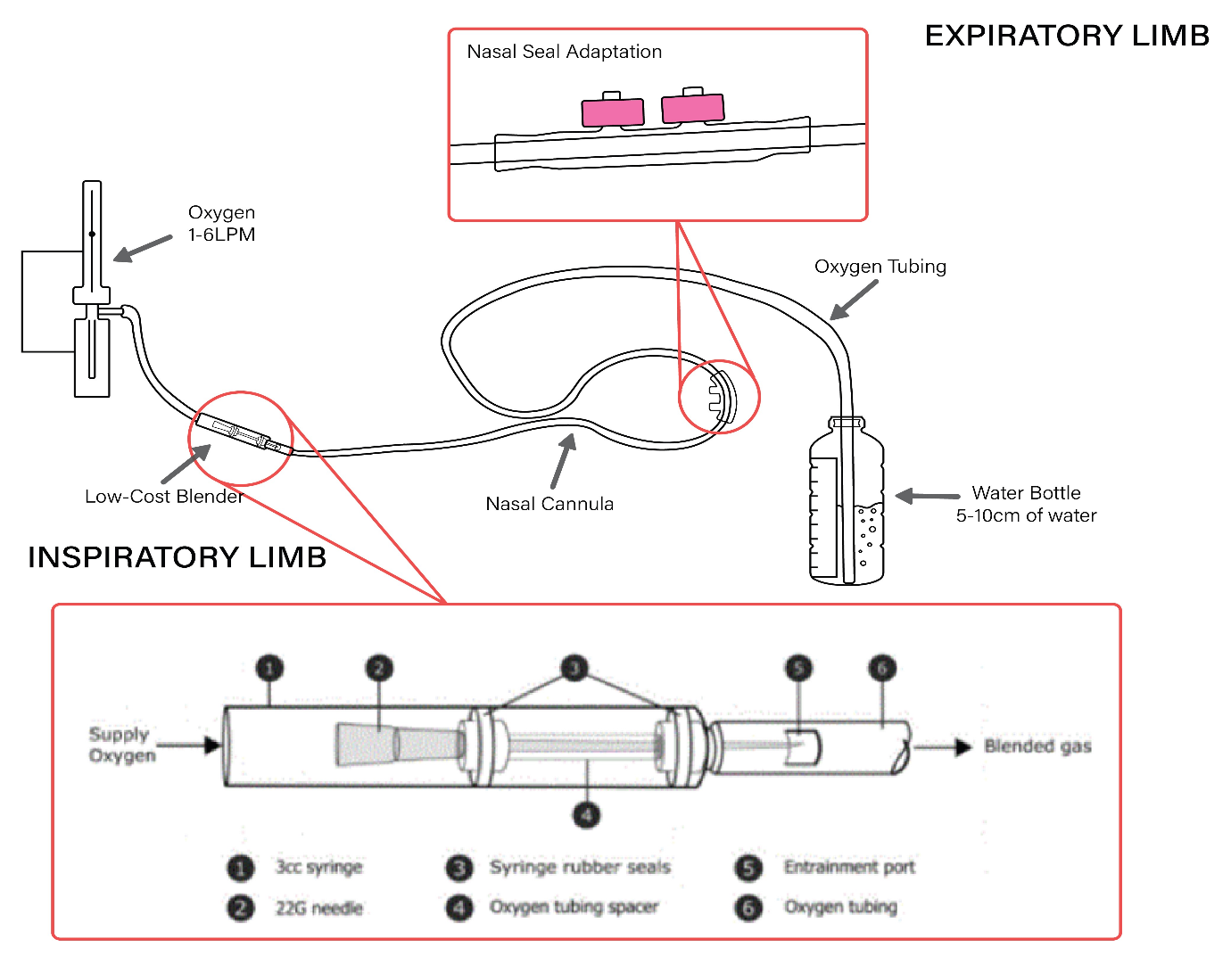


Supplies Needed

1. 3cc Syringe (2x of the same brand) with rubber stoppers on the plungers
2. 22 gauge hypodermic needle
3. Nasal cannula (adult or peds)
4. Tape
5. Knife or scalpel
6. Oxygen connector tubing with adapter on both ends
7. Super glue with fine tip applicator (if available)

Instructions for Assembly

1. Begin with the nasal cannula (c). Open the package and cut along the red line as shown below.


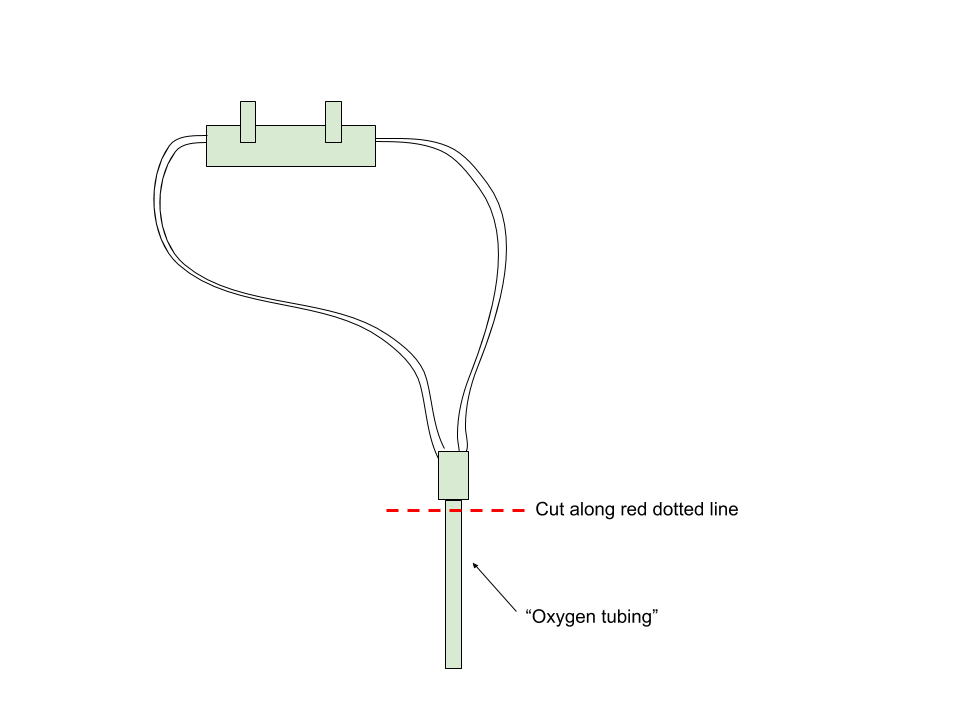


1. Gather two 3cc syringes (a) of the same brand. Remove the plunger from both. After removing both plungers, pry the rubber stopper off of both plungers.


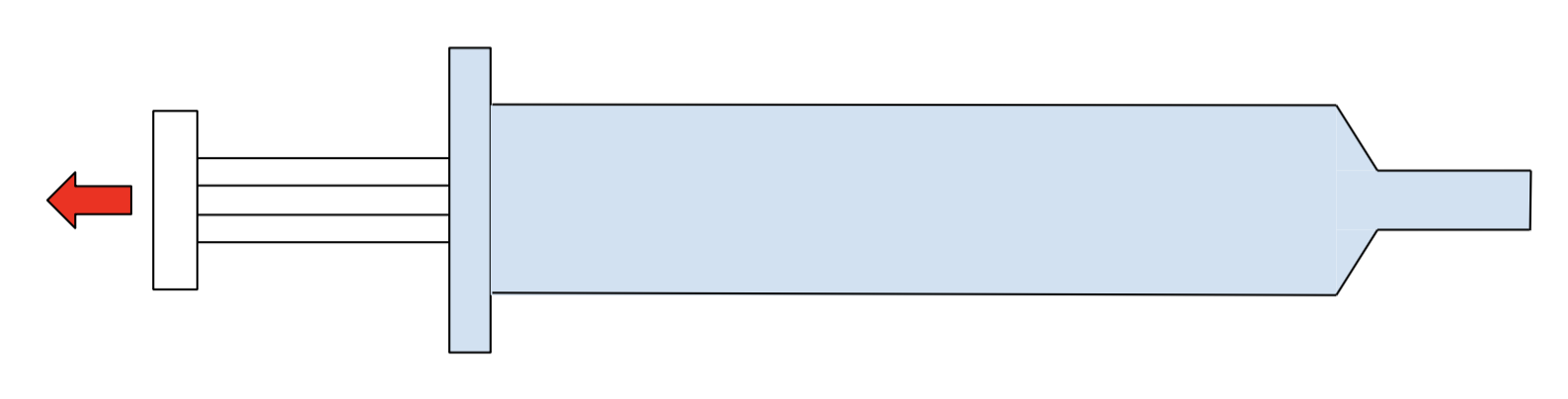


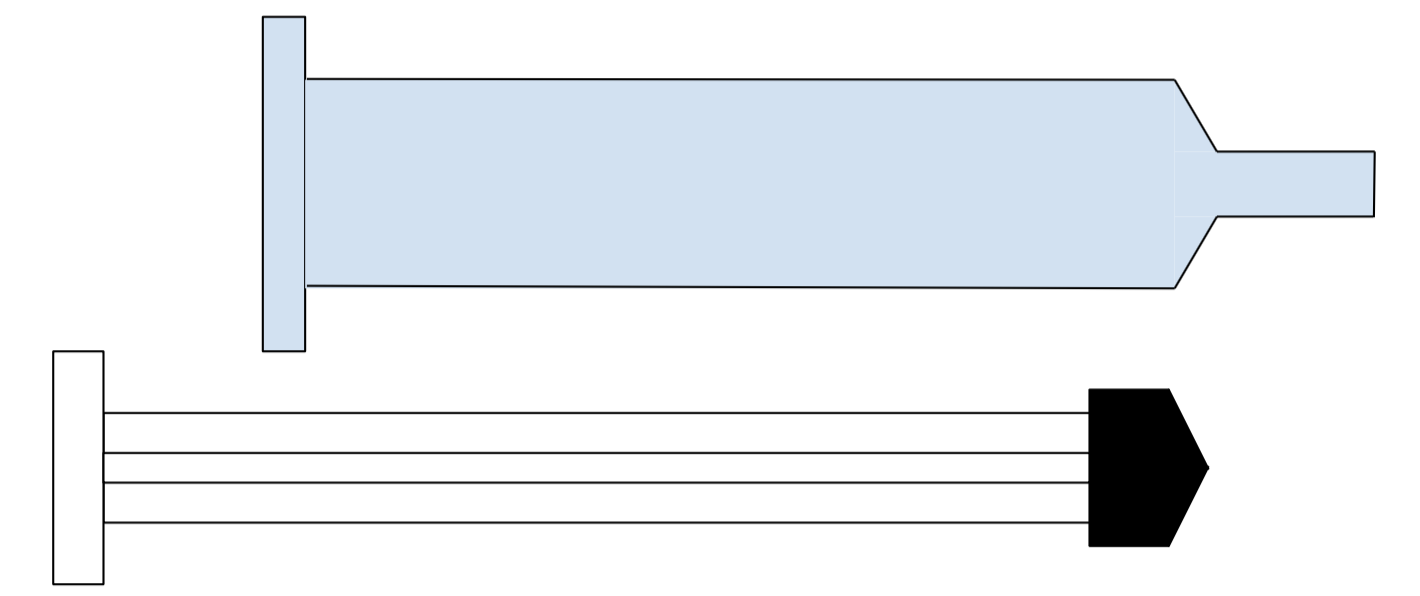


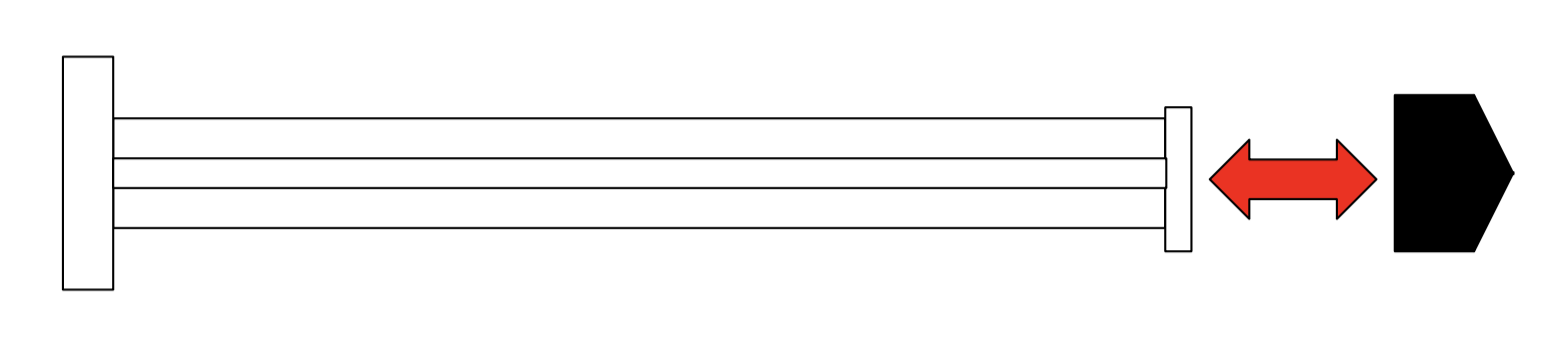


1. Take a 22 gauge needle (b) and the rubber stopper (a). Stab the 22 gauge needle through the back of the rubber stopper, **as close as you can to the center.** If your stick is not perfectly centered, it is OK to remove the needle and try again as the rubber is self-healing. **Make sure the sides of the rubber stopper are very close to parallel with the needle.** Once the rubber stopper is skewered by the needle, slide the rubber stopper all the way down to the needle’s base.


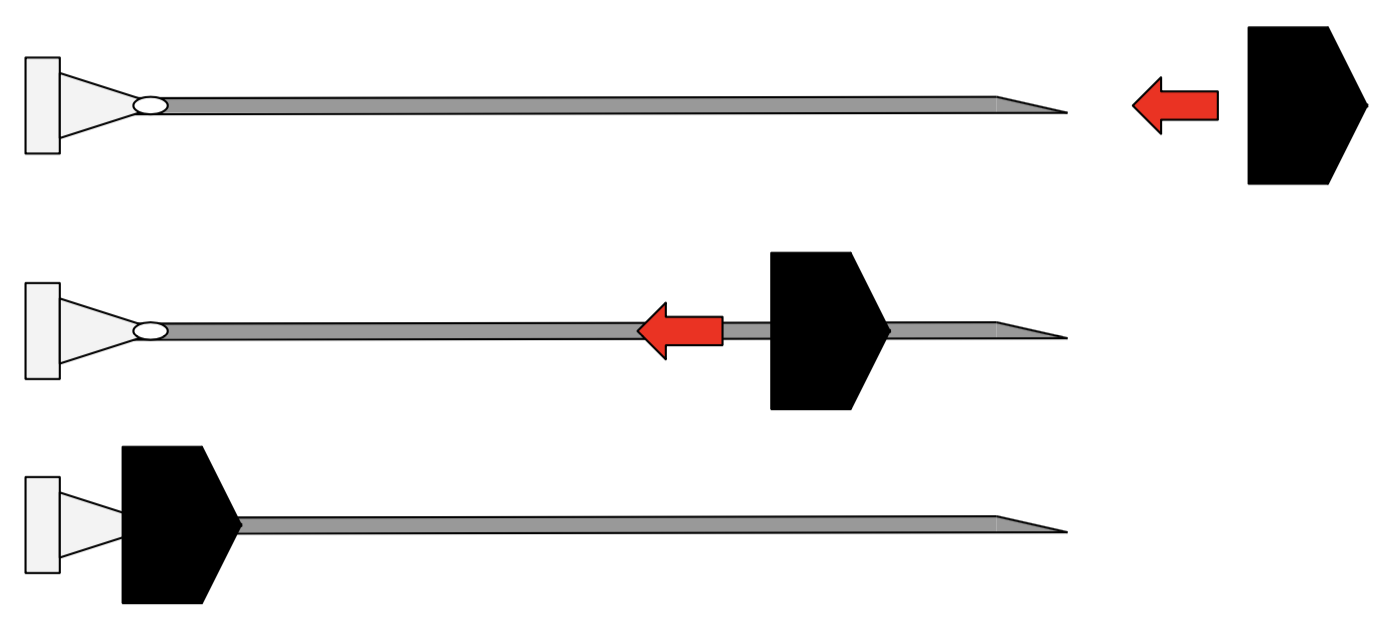


1. Cut a length of oxygen tubing (c) obtained in Step #1 to be equal to the ‘spacer length’. This part is called the ‘spacer’. **For a 1.5 inch 22 gauge needle, this length should be 1.5 cm. For a 1 inch needle, this length should be about 0.5cm.** The needle should not stick out too far out of the syringe. If it does, it may move when flow is turned on.


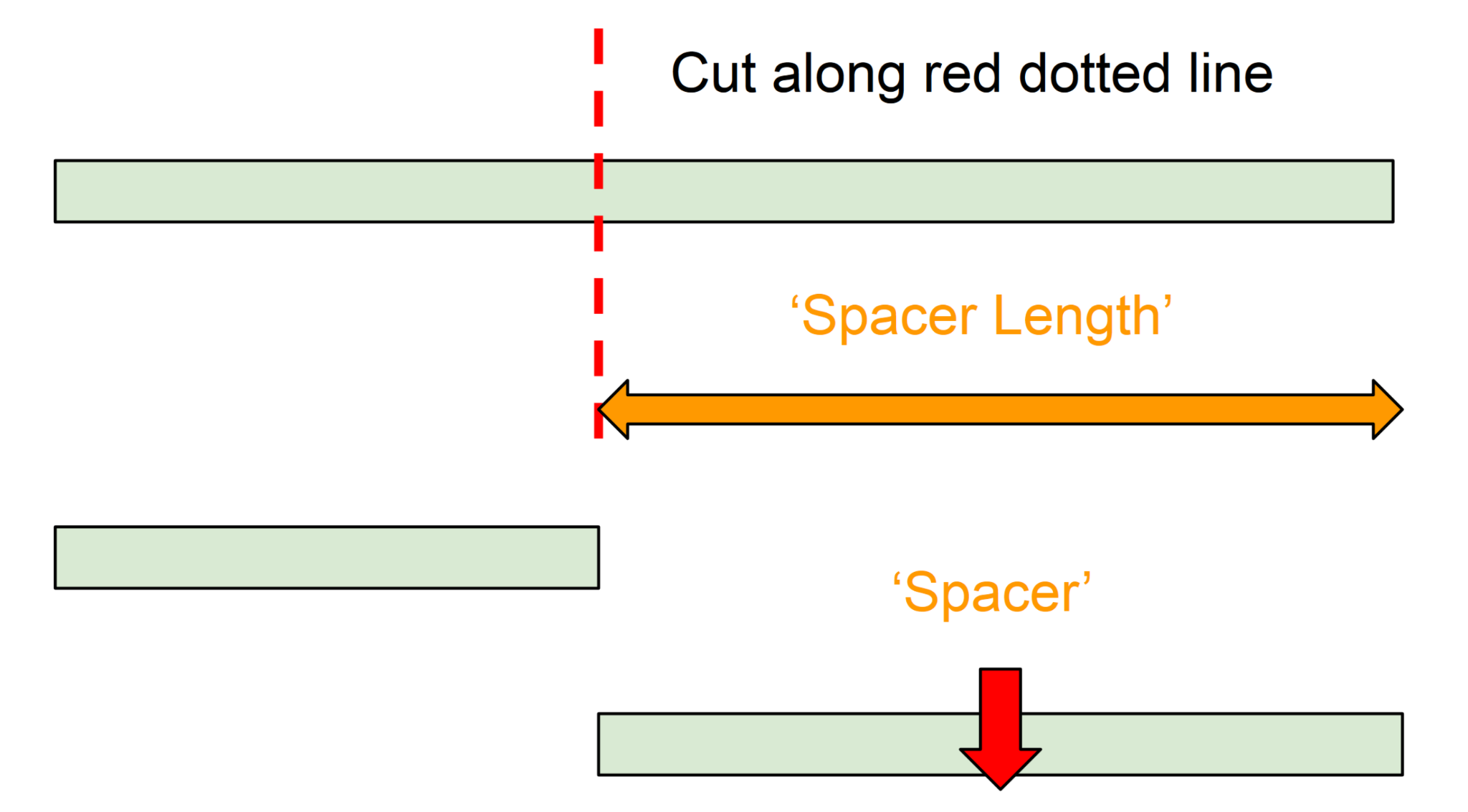


1. Slide the ‘spacer’ over the needle, so that it rests on the rubber seal towards the base of the syringe. Put the rubber stopper back on, and slide it so that the rubber stoppers ‘sandwiches’ the ‘spacer’. This assembly is called the ‘blender internals’.


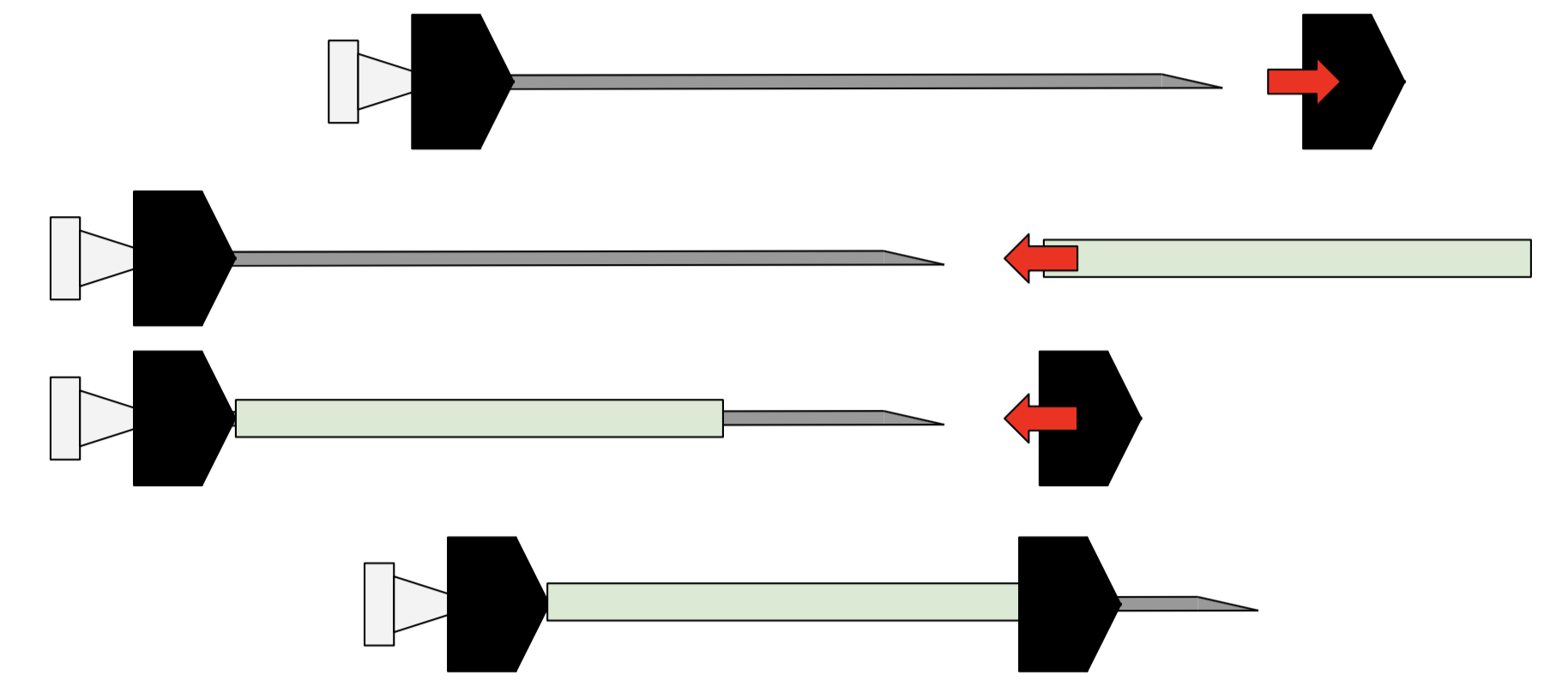


1. Insert the blender internals into one 3cc syringe (a), with the tip of the needle going first. Use one plunger from the 3cc syringe to push it all the way down the syringe barrel, so that it ‘bottoms out’. **It will be difficult to re-insert the plunger into the syringe. To do this more easily, insert the plunger at an angle initially, and slowly push it in.**


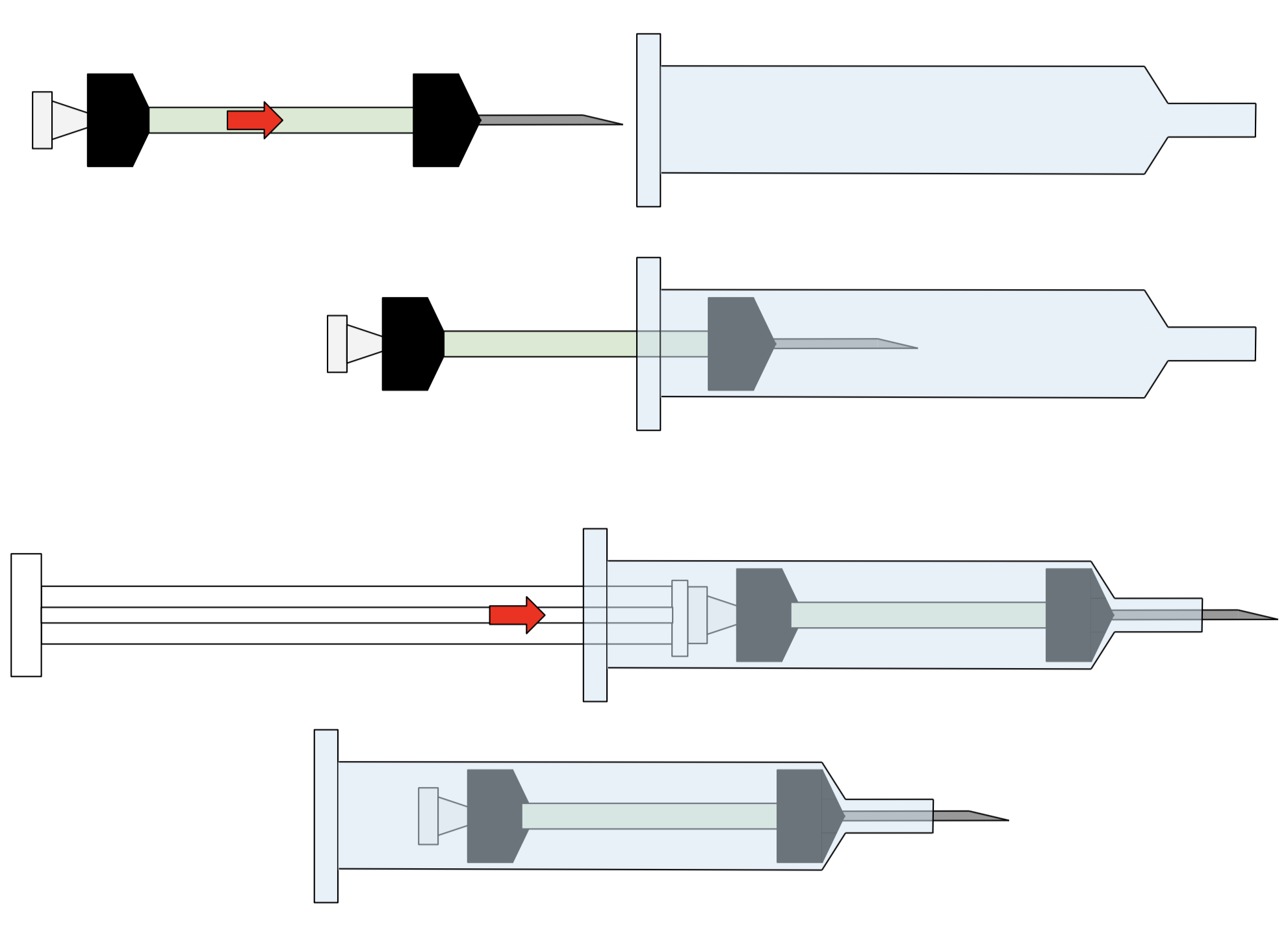


1. Measure the internal diameter of the 3cc syringe (a). Record this number. Take the oxygen connector tubing (f). Cut the connector tubing so that the largest outer diameter of the tubing is 2mm larger than the internal diameter of the 3cc syringe. Ensure that the oxygen connector can be inserted into the 3cc syringe if you press hard enough.


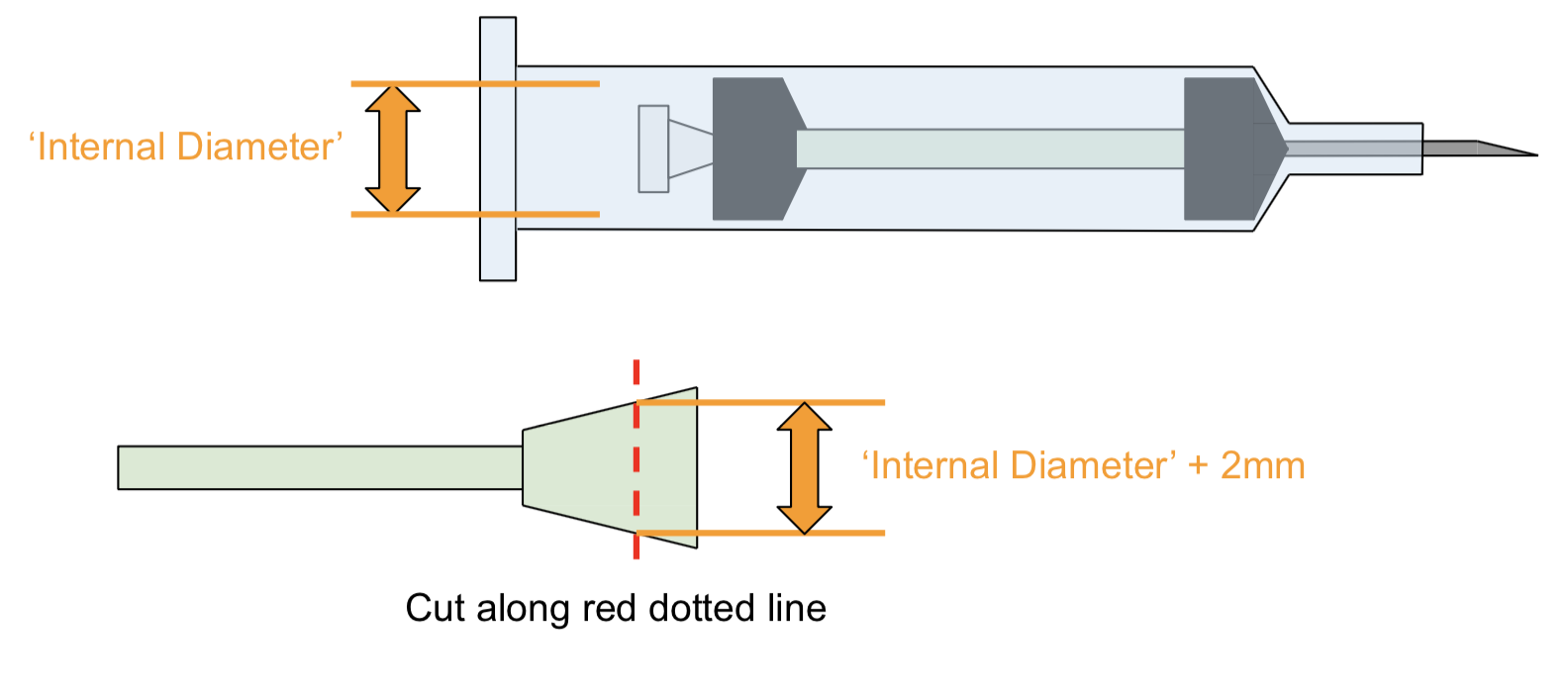


1. Place superglue on the exterior part of the oxygen connector and insert it into the 3cc syringe body.
   1. Alternatively, tape can be used. Insert the oxygen connector into the 3cc syringe and tightly tape them together, using medical tape, electrical tape, or duct tape.


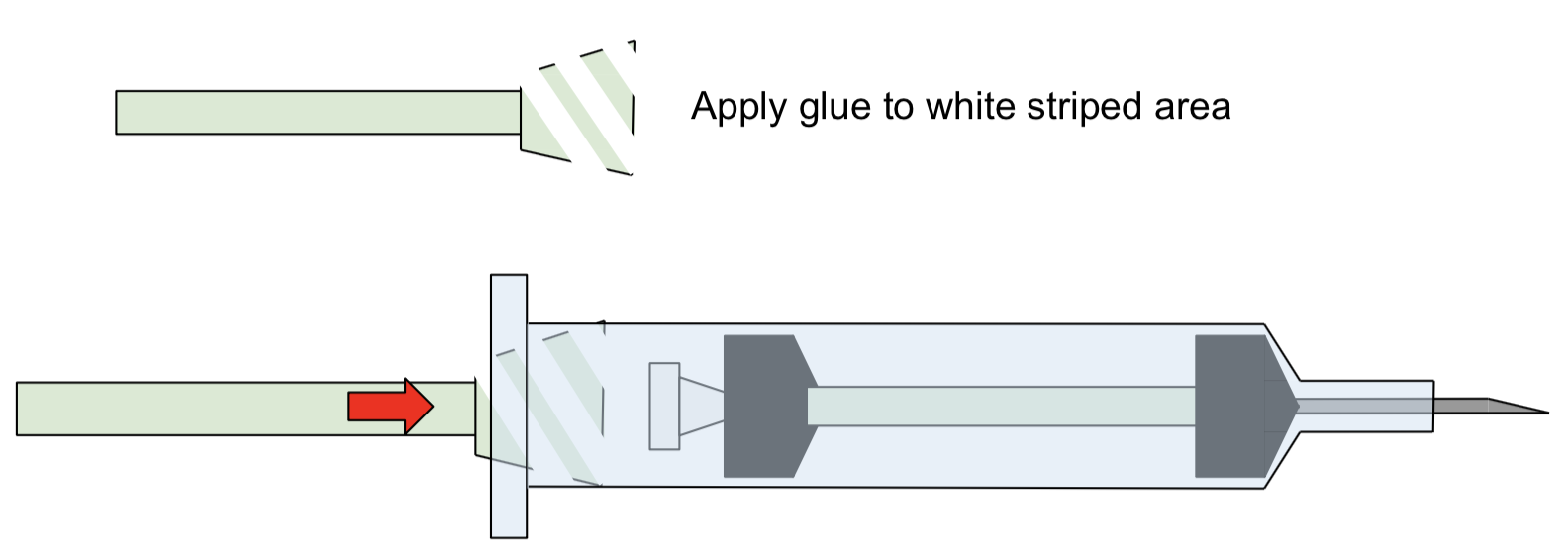


1. Take the 3cc syringe (a) and insert the luer end into the oxygen tubing (c) prepared earlier in Step #1.


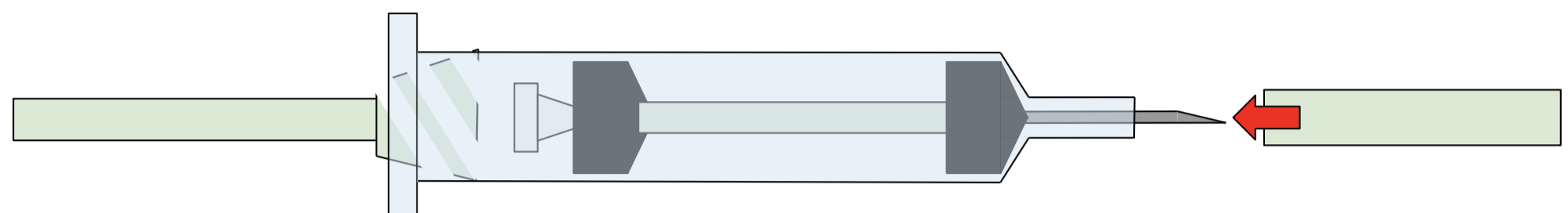


1. Now, a small port will be made to allow air to flow into the oxygen tubing. Make a slice in the oxygen tubing directly above the end of the needle tip. **This slice should be perpendicular to the needle, and should go approximately halfway through the tubing.** Measure 5mm or 10mm away from the needle tip **(Note: use 5mm if a 65% oxygen blender is desired, or 10mm if a 50% oxygen blender is desired).** Make another slice in a similar manner, again going halfway through the oxygen tubing. Connect these two slices with cuts running parallel to the needle, so that a small rectangle can be removed from the tubing, as shown below. This hole is referred to as the ‘port’.


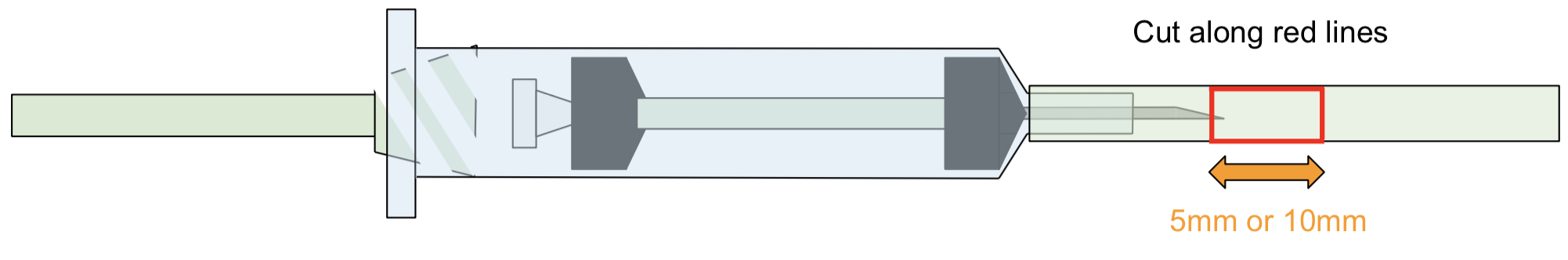


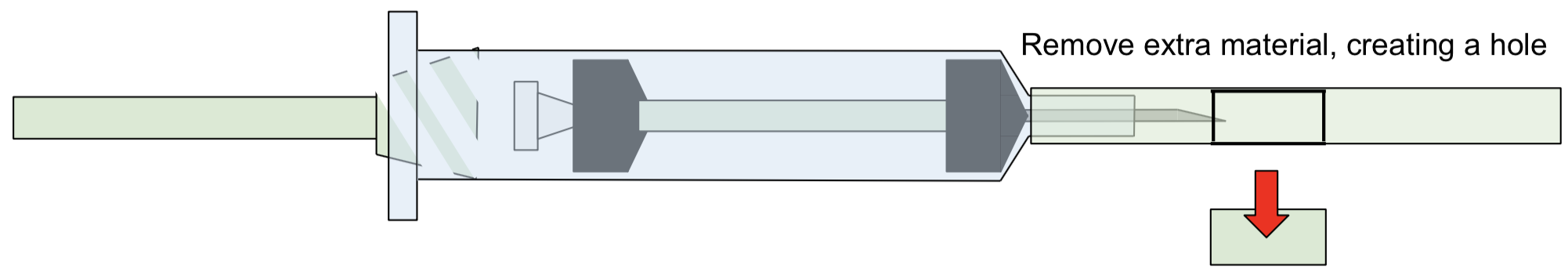


1. Tape the oxygen blender down to a table or tongue depressor. This is best accomplished by first taping the 3cc syringe itself down, followed by the tubing connecting to the oxygen blender. Be sure that the tape on the oxygen tubing is placed at least 5cm away from the port and that it **does not bend the port downwards.**


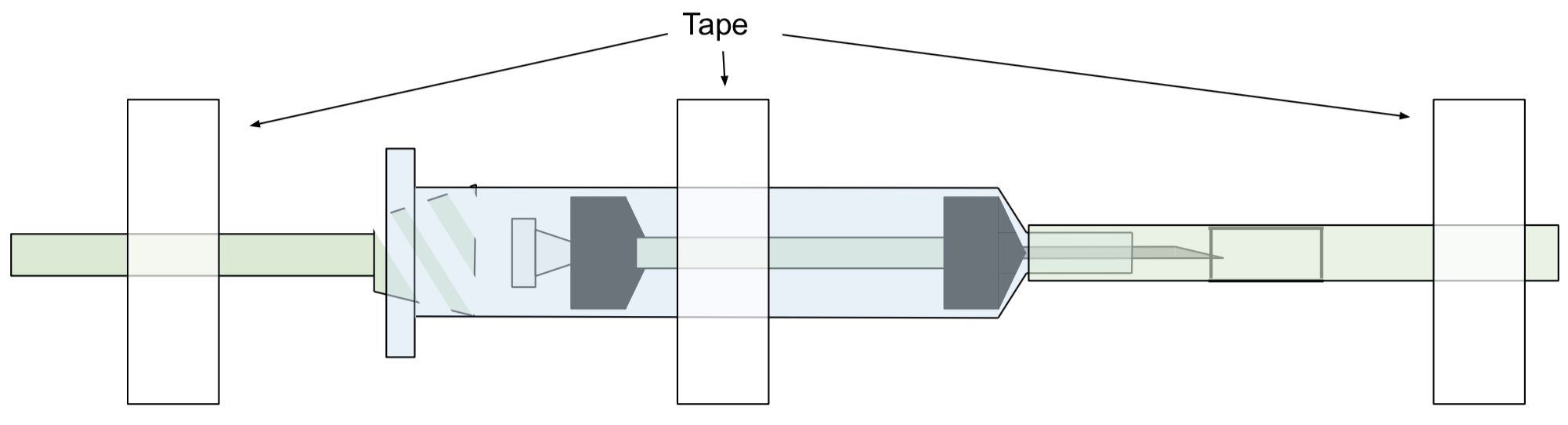


1. In order for the blender to operate correctly, a modified cannula must be created. To do this, take the nasal cannula modified earlier in Step #1. Cut the cannula as shown in the picture below, make sure that the cannula is cut as close as possible to the sections where the diameter changes.


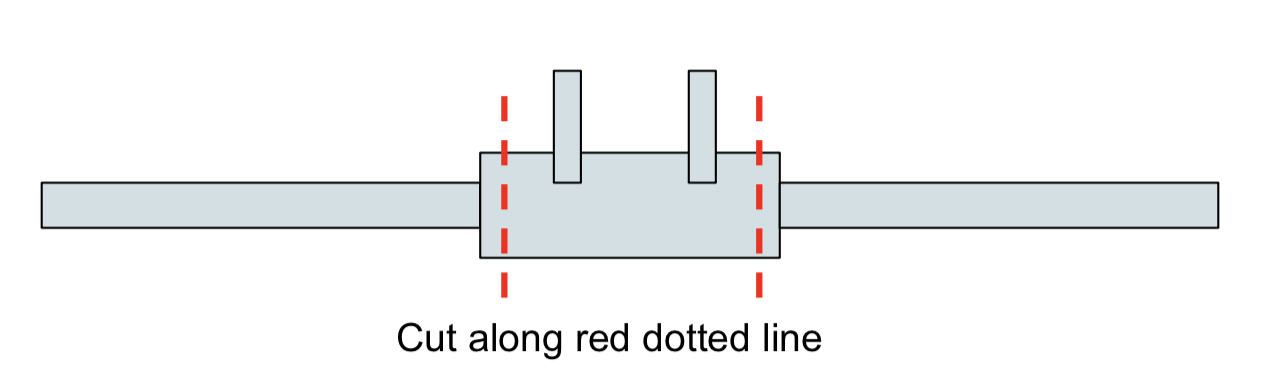


1. Make a slit running along the bottom of the cannula, cutting through the material completely.


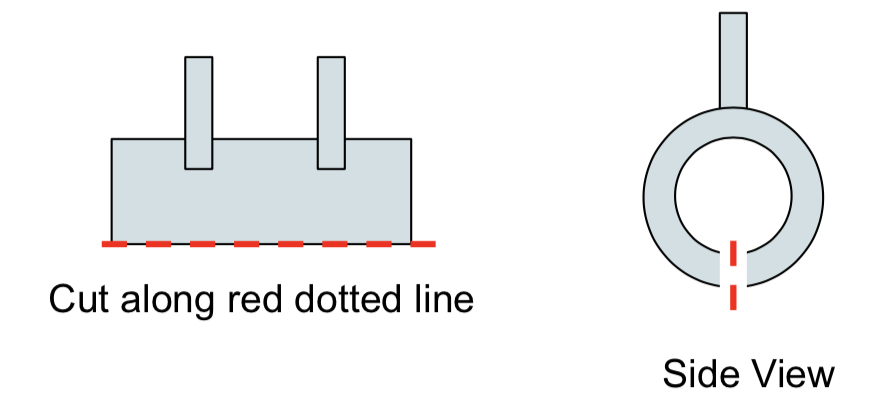


1. Line up the cannula alongside **the downstream section** **of oxygen tubing (i.e. tubing with the port),** about halfway between the end and where it connects to the oxygen blender and along the inside of the natural curve of the tubing. Make a mark on the oxygen tubing immediately ‘outside’ the width of the cannula nasal prongs, as shown below. Cut a 5mm wide rectangle in the oxygen tubing spanning this width, in the same manner that was used before in Step #10. Remove excess material.


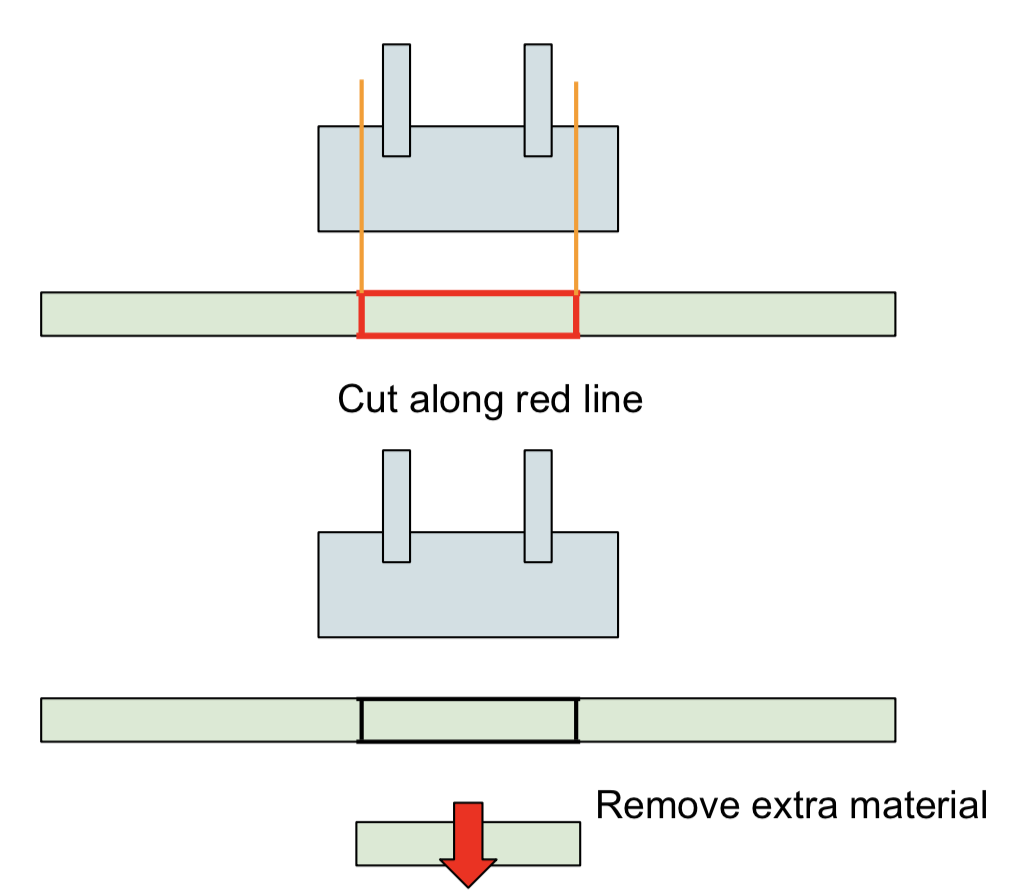


1. Wrap the cannula around the hole created in the oxygen tubing. Make sure there are no exposed holes.


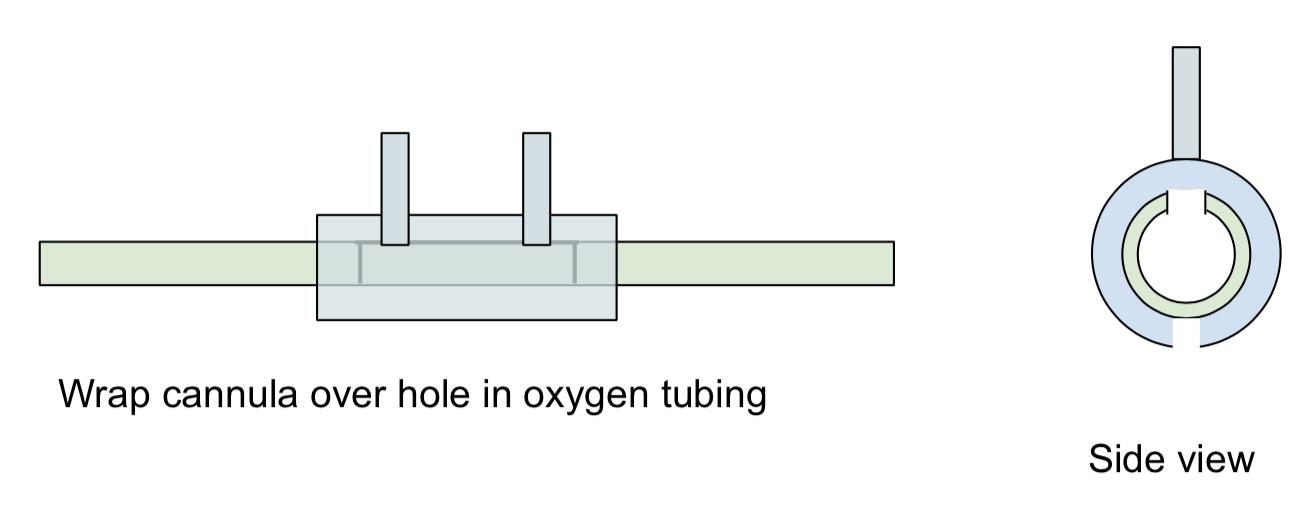


1. Tape the cannula down at both ends. Then, tape along the bottom. Add tape as needed so that there are no holes, but do not tape between the cannula prongs to minimize nasal abrasions.
2. Take the remaining downstream end of the oxygen tubing and place it in a water bottle to the desired CPAP level. Insert the cannula into the patient’s nostrils. Attach the free end of the oxygen connector tubing (f) to the oxygen source. Increase the flow rate **slowly** on the oxygen tank until a steady, gentle stream of bubbles exits the water bottle. The LESS O_2_ blender is now complete & operational.

Instructions for Use

| **Indication for use** | The LESS O_2_ blender is intended to be used for any patient under 5 years of age who is in respiratory distress and where low flow nasal cannula or WHO-style bubble CPAP with 100% oxygen are the only alternatives [1].  The ideal patient is a child less than 8 months old with mild to moderate respiratory distress from a lower respiratory tract infection. |
| --- | --- |
| **Changing the oxygen level (i.e. FiO_2_)** | To increase from ~50% to ~65%, change circuit with 10mm port to a circuit with a 5mm port.  To increase to ~100%, you must discontinue the blender circuit and place on WHO-style bCPAP with no blender [1].  To decrease from ~50% to ~65%, cut tubing to make entrainment port longer (from 5mm to 10mm in length). |
| **Changing the flow of oxygen** | There is no set flow. Increase flows as needed to achieve gentle bubbling.  Do not increase oxygen flow to more than 6LPM as this may compromise the blender and lead to fragmentation. If lower flows do not achieve bubbling, check for leaks (see “Troubleshooting” below on page 13). |

Preventing Complications

| **Nasal Injury** | Septal injury is preventable with *frequent monitoring and prong repositioning*. It is not a reason to discontinue using the nasal prongs.  Evaluation of the nasal septum should be done at least every 6 hours or more frequently (damage can occur even after a few hours of applied pressure).   1. __Use the correct prong size. 2. __Keep skin dry 3. __Secure the prongs in place so that they are not moving 4. __Do not allow the bridge of the prongs to press up against the septum. 5. __Avoid twisting the prongs with resultant lateral pressure against the septum. |
| --- | --- |
| **Shifting of Blender Internals** | Sometimes when the flow is changed too quickly, the blender internals can shift inside the syringe. This usually means the seal is very tight (i.e. no leaks), but can cause the blender to malfunction *if the needle comes back too far and does not move forward when turning on oxygen flow.*  Always change the flow slowly – whether increasing or decreasing the flow. |
| **Fragmentation of the Blender** | This is a very high pressure system. You may have to use a significant amount of tape to keep tubing together (3-4 wrap arounds). Use glue with a fine tip applicator if available.  If using 6LPM, apply glue to the connection between tubing from the oxygen tank to the syringe. Check the system 30 minutes after starting the flow at 6LPM. Reduce flow as soon as possible to prevent fragmentation. |

Troubleshooting

| **If no bubbles are present at all or only intermittent (i.e. not continuous)** | The patient is **NOT** getting effective CPAP.  __Check the patient for stability  __Make sure the prongs are in the nose. Check for bubbling  __Occlude the nasal prongs with your fingers.  If there is bubbling, the problem is a nasal seal or the patient’s mouth is open.  __Temporarily close the patient’s mouth. If bubbling occurs, soothe patient if crying or use a hat with a chin strap to close mouth  __Use larger nasal prongs  __Use ear plugs (SEAL-bCPAP) to ensure nasal seal around prongs [2]  If no bubbling, there is a leak in the circuit. **The most common places for leaks** are  __the nasal prongs taped/glued to the oxygen tubing  __the connection between the oxygen adapter tubing and the adapter  __the connection to the oxygen tank  __the connection between the syringe and the oxygen tubing from the tank.  __Unhook the syringe from the downstream oxygen tubing. Place the syringe/needle in water with the oxygen on. If there is bubbling, the leak is occurring after the syringe. If there is no bubbling, the leak is before the syringe.  If you suspect a leak, try sealing with your fingers to see if that generates bubbles. If it does, apply tape or glue as needed to seal the found leak.  Check the syringe needle and port  __Check that the port is not twisted or not bent left or right or up or down. You may manipulate the tube to see if bubbles are created.  __If bubbles are **intermittent**, cover the entire entrainment port with your finger. If using a 5mm port, the bubbling strength should decrease. If using a 10mm port, the bubbles should not change. If bubbles **greatly** increase, there is a leak in the port and the device needs to be rebuilt.  __Make sure the needle ends exactly where the port begins. If not, cut off the port and create a new one  If all else fails, turn the flow up to see if able to overcome any leak/resistance that may be present  Lastly, you can replace the entire tubing system. You may also re-use the blender internals by removing them from the syringe with curved forceps. |
| --- | --- |
| **If nasal injury is present** | Nasal injury appears as redness or irritation of the nasal septum, nostrils (nares), or skin surrounding the nose where the prongs have been pressing.   1. __If signs of erythema or erosion are observed, reposition the prongs to remove the pressure, friction, or moisture. |
| **If the blender internals have moved backwards** | If the plunger has moved backwards into the syringe:  __Increase the oxygen flow until the needle moves forward to where it was before. Then slowly decrease the flow to the desired rate.  __Remove the tubing from the back of the syringe and use a plunger to push the internals through again.  __If the internals continue to move backwards, remove the internals with curved forceps and reconstruct OR build a new device OR use a new blender. |
| **If the blender fragments** | If fragmentation occurs:  __Ensure the patient is stable  __Turn off oxygen source (tank)  __Place patient on WHO-style bCPAP [1]  __Check blender internals for shifting  __Reseal with tape or glue and slowly increase oxygen flow  __If using glue, apply and wait 15 seconds before turning the flow back on. |

References

1. *Oxygen therapy for children: a manual for health workers*, ed. W.H. Organization. 2016, Geneva: World Health Organization.

2. Bjorklund, A.R., et al., *Use of a modified bubble continuous positive airway pressure (bCPAP) device for children in respiratory distress in low- and middle-income countries: a safety study.* Paediatr Int Child Health, 2019. **39**(3): p. 160-167.

Contact and Support

If you have any questions, comments, or feedback regarding the device, please email the corresponding author at [andrew.wu@hcmed.org](mailto:andrew.wu@hcmed.org). The authors would particularly appreciate any feedback regarding complications or suggestions for improvement. Thank you for all you do.
